# Cellular interactions and evolutionary origins of endosymbiotic relationships with ciliates

**DOI:** 10.1093/ismejo/wrae117

**Published:** 2024-06-25

**Authors:** Qi Song, Fangqing Zhao, Lina Hou, Miao Miao

**Affiliations:** Medical School, University of Chinese Academy of Sciences, No. 1 Yanqihu East Road, Huairou District, Beijing 100049, China; Medical School, University of Chinese Academy of Sciences, No. 1 Yanqihu East Road, Huairou District, Beijing 100049, China; Institute of Zoology, Chinese Academy of Sciences, 1 Beichen West Road, Chaoyang District, Beijing 100101, China; Key Laboratory of Systems Health Science of Zhejiang Province, School of Life Science, Hangzhou Institute for Advanced Study, University of Chinese Academy of Sciences, No. 1 Xiangshan Road, Hangzhou 310024, China; Medical School, University of Chinese Academy of Sciences, No. 1 Yanqihu East Road, Huairou District, Beijing 100049, China; Medical School, University of Chinese Academy of Sciences, No. 1 Yanqihu East Road, Huairou District, Beijing 100049, China

**Keywords:** acquired phototrophy, algae, ciliates, endosymbiosis, host-bacteria interaction

## Abstract

As unicellular predators, ciliates engage in close associations with diverse microbes, laying the foundation for the establishment of endosymbiosis. Originally heterotrophic, ciliates demonstrate the ability to acquire phototrophy by phagocytizing unicellular algae or by sequestering algal plastids. This adaptation enables them to gain photosynthate and develop resistance to unfavorable environmental conditions. The integration of acquired phototrophy with intrinsic phagotrophy results in a trophic mode known as mixotrophy. Additionally, ciliates can harbor thousands of bacteria in various intracellular regions, including the cytoplasm and nucleus, exhibiting species specificity. Under prolonged and specific selective pressure within hosts, bacterial endosymbionts evolve unique lifestyles and undergo particular reductions in metabolic activities. Investigating the research advancements in various endosymbiotic cases within ciliates will contribute to elucidate patterns in cellular interaction and unravel the evolutionary origins of complex traits.

## Introduction

Symbiosis, a phenomenon observed across distinct species, has manifested in various forms in nature. These associations are recognized as significant driving forces of ecological functions and evolutionary processes, fostering interdependence and coevolution [1–5]. Scenarios involving the origins of eukaryotic organelles (e.g. mitochondria and chloroplasts) through early endosymbiotic events have been widely accepted, although ongoing debate persists regarding the initial establishment and subsequent metabolic evolution [6–8]. In some cases, one partner in this relationship may benefit at the expense of the other, a phenomenon known as parasitism [1, 9, 10]. Conversely, when one partner is minimally affected, the interaction is termed commensalism [9, 10]. Alternatively, in scenarios where both symbiotic partners derive benefits from their collaboration, the relationship is categorized as mutualism [9–11]. Furthermore, symbiotic relationships can be further categorized based on the differing nutritional functions and levels of interdependence between partners into obligate and facultative symbiosis, although the delineation between these categories may blur under specific circumstances [12, 13].

The unicellular protozoan **ciliate** (see Glossary) stands out as a prominent member of microbial predators, thriving in diverse environments [14, 15]. Renowned for its complex structures and functions, the ciliate serves as a valuable model for cytology, ecology, and genetic studies [16, 17]. Ciliates, through the phagocytosis of prey, create opportunities for potential colonization outside host **digestive vacuoles (DVs)**, either actively or passively [18, 19]. Subsequent energy transmission and substances exchange with the host can lead to the establishment of symbiotic relationships [20].

Phagotrophic organisms that harbor and exploit phototrophs or photosynthetically active organelles are considered to have **acquired phototrophy**, transitioning into a state of **mixotrophy** [21, 22]. Acquired phototrophy events are widespread among planktonic protists in fresh waters, estuary areas, coastal waters, and open oceans [23–26]. Protists in these environments exhibit varying degrees of phototrophy dependance, challenging the traditional categorization of these organisms as consumers or producers, and complicating the trophic structure of certain ecosystems [4, 23, 27, 28]. Mixotrophy play a significant role in the redistribution of carbon biomass to higher tropic levels and the augmentation of sinking carbon flux [4, 29]. Many *Foraminifera* species acquire photosynthates from symbiotic algae, with certain consortia emerging as prominent members in oligotrophic environments, boasting substantial primary production [30–32]. Dinoflagellates also exhibit various patterns of acquired phototroph: some harbor phototrophic algae (e.g., *Noctiluca scintillans* bearing thousands of green *Pedinomonas noctilucae*), while others sequester algal chloroplasts (e.g., *Dinophysis* spp. exploiting cryptophyte chloroplasts) [33–35].

Endobacteria, characterized by high diversity, are prevalent in numerous documented endosymbiotic relationships [36, 37]. These adaptable bacterial symbionts inhabit various compartments within host cells, where specific selective pressures have led to extreme reductions in genome size and streamlined metabolism [38, 39]. While essential for the survival of obligate symbiotic bacteria, these relationships are not always indispensable for protists, exhibiting a spectrum from mutualistic to parasitic, potentially conferring benefits or harm to hosts in specific contexts [40–42]. Some soil-dwelling social amoeba, such as *Dictyostelium discoideum* farmer clones, have been observed to host symbiotic *Burkholderia* bacteria, which aid in promoting host growth and inhibiting non-farmer clone competitors by secreting biomolecules [43, 44]. Additionally, amoebae serve as environmental hosts for the human pathogen *Legionella pneumophila*, capable of releasing effector proteins to modulate host cell metabolism and cellular processes for their own advantage [44, 45].

Unlike multicellular hosts, where symbiont distributions are typically confined, ciliates engage in symbiotic relationships throughout their entire body, providing a distinctive perspective and serving as an ideal model system for investigating the mechanisms and evolution of intracellular endosymbiosis. This review focuses on the current understanding of representative patterns of acquired phototrophy and specific endobacteria phenomena observed in ciliates within freshwater and oceanic ecosystems. Emphasis is placed on the establishment, maintenance, and physiological properties of ciliate endosymbiosis. Our work delves into representative reported cases, aiming to illustrate the diverse forms of mixotrophic evolution between ciliates and planktonic microorganisms including algae and bacteria.

## Ciliates and intact algal symbionts

Multiple instances of acquired phototrophy are exemplified by intracellular phototrophic organisms, with zoochlorellae and zooxanthellae predominately occupying freshwater and marine environments, respectively. These algae are engulfed entirely by the host and maintain their original structure and reproductive capability.

**Figure 1:**
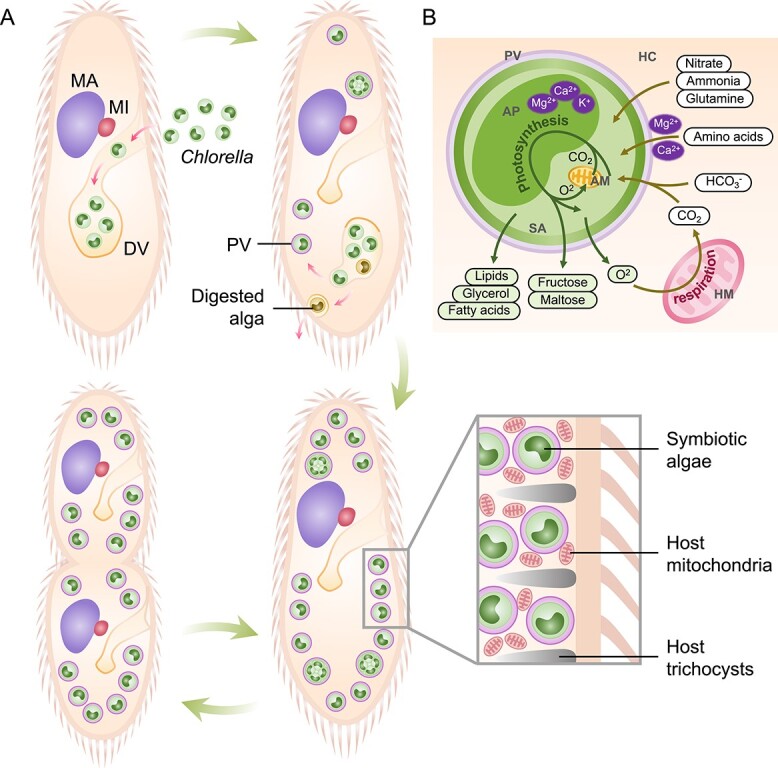
**Illustration of reinfection and metabolic interaction between *Paramecium* and symbiotic *Chlorella*.** (A) The reinfection process of *Chlorella* in *P. bursaria*. Algae-free *P. bursaria*, when mixed with *Chlorella*, allows algae entry via phagocytosis in DVs. After a series of digestion processes, surviving algae maintain their original green color, while others turning yellow or brown are digested or expelled. Surviving algae escape DVs through membrane budding, and DV membranes evolve into specific PV membranes, shielding algae from lysosomal system digestion. Escaped algae move beneath the host cell surface, undergo division, attach to the cell cortex, with the host trichocysts dispersing aside. Host mitochondria connect to PV membranes, forming a network linked to the host endoplasmic reticulum. Symbiotic algae are distributed to both daughter host cells during division. (B) Schematic representation of the main predicted metabolic interactions between *P. bursaria* and *Chlorella*. Green arrows indicate the release of organic matter and O_2_ by endosymbiotic *Chlorella* for host cell utilization. Brown arrows represent host conversion of O_2_ into CO_2_, translocated into algal cells along with other carbon sources, nitrogen sources, and amino acids. Purple circles denote host factors regulating photosynthesis and amino acid uptake. MA, macronucleus; MI, micronucleus; DV, digestive vacuole; PV, perialgal vacuole; HC, host cell; SA, symbiotic alga; AP, algal plastid; AM, algal mitochondrion; HM, host mitochondrion.

*Paramecium bursaria,* a well-known aerobic free-swimming ciliate, is recognized for its symbiotic association with algae [46, 47]. Each cell of *P. bursaria* typically hosts hundreds of symbiotic algae in its cytoplasm. Various algae, such as *Chlorella variabilis*, *Micractinium conductrix*, and the recently confirmed *Choricystis parasitica*, have been discovered within the cytoplasm of *P. bursaria* [48, 49]. Among these symbiotic algae, the unicellular zoochlorellae *Chlorella* has been extensively studied due to its widespread association with ciliates in freshwater habitats. The establishment of the *Paramecium*-*Chlorella***symbiosis** mainly involves the phagocytosis of algae, budding from DVs, and colonization of the host cytoplasm (Fig. 1A) [50–53]. During this process, the algae’s ability to resist digestion plays a critical role. It has been observed that this resistance is independent of heredity, growth stages, or the location within host DVs [18, 54]. However, constant dark treatment noticeably reduces algae’s survival rate [18]. The surviving algae then bud from DVs, and the DV membrane differentiates into the **perialgal vacuole** (PV) membrane, a process found to be related to vacuole contents sizes [51–53, 55, 56]. Dynamin has been implicated in the budding of DV membranes, as evidenced by the significant inhibition of DV budding observed with its inhibiter, dynasore, leading to the absence of isolated algae in the cytoplasm [55]. Subsequently, the released algae migrate to the underside of the host cell cortex, displacing pre-existing trichocysts and establishing contact with host mitochondria [53, 57, 58].

Algae enclosed in PVs beneath the host cell cortex can undergo cell division (Fig. 1A) [52, 59, 60]. Typically, each alga cell division results in the formation of two or four autospores, with the PV membranes expanding synchronously [60]. After division, the enlargement of daughter cells leads to the fragmentation of the mother cell wall, which is subsequently expelled [59, 60]. The daughter cells are then individually encapsulated within PV membranes [59]. Symbiotic algae have been reported to double their population to synchronize with host cell division, maintaining a nearly constant level throughout the host cell cycle [61, 62].

Under sufficient illumination, the majority of *Chlorella* cells within *P. bursaria* exhibit a green coloration and demonstrate a high growth rate. These algae benefit from the inorganic nutrients, such as nitrogen and CO_2_, provided by the host’s metabolism, reciprocating with the supply of photosynthate, including oxygen and maltose (Fig. 1B) [63–65]. This symbiotic exchange enables *P. bursaria* to be relatively independent of external food provision. Even during periods of starvation, *P. bursaria* can extend its survival duration through the digestion of symbiotic *Chlorella* [66]. Symbiotic digestion additionally provides energy for the biosynthesis of trichocysts, which are utilized during starvation [66]. Additionally, the shelter of *P. bursaria* provides *Chlorella* with protection against negative influences from potential competitors (e.g., *Chlamydomonas reinhardtii*) or infection by *Chlorella* virus [67, 68].

While both *P. bursaria* and *Chlorella* benefit from this symbiotic relationship, they can still lead independent lives. Various approaches, including treatment with protein synthesis inhibitor cycloheximide, photosynthesis inhibitor 3-(3′,4′-dichlorophenyl)-1,1-dimethylurea (DCMU), or cultivation in constant darkness, can induce algae-free *P. bursaria* [18, 69, 70]. Algae-free *P. bursaria* cells are smaller in size compared to algae-bearing cells, and their proliferation rate significantly weakens [67, 69, 71]. The presence of endosymbiotic *Chlorella* also significantly reduces the number of host mitochondria, accompanied by a lower content of mitochondrial proteins compared to algae-free host cells [72]. Notably, separated *P. bursaria* and *Chlorella* can readily form new symbiotic combinations by mixed together.

Differential transcriptome analysis reveals the up-regulated expression of heat shock 70 kDa protein (Hsp70) in algae-bearing *P. bursaria* compared to algae-free cells, potentially explaining their higher survival rate under high temperatures [73, 74]. RNA interference experiments, based on differentially expressed genes between algae-bearing and algae-free *P. bursaria*, suggest that glutamine may play an essential role in maintaining symbiosis with *Chlorella* [71]. Intriguingly, *P. bursaria* exhibits fewer genes involved in oxygen binding compared to closely related species, likely attributed to the long-term coevolution with endosymbiotic *Chlorella* [71].

Zoochlorellae also engage in symbiotic relationships with other freshwater ciliate species. *Chlorella* has been identified in *Euplotes daidaleos*, *Climacostomurn virens*, *Stentor polymorphus*, *Stentor araucanus*, and *Stentor pyriformis* [75–78]. *Stentor polymorphus*, for instance, can host algae of the genus *Mychonastes* as symbionts, and the photobionts in *Stentor amethystinus* were identified as *Coccomyxa* sp. [77, 79]. Additionally, *Frontonia vernalis*, *Halteria viridis*, *Ophrydium versatile*, and *Ophrydium naumanni* also harbor symbiotic zoochlorellae, although the algae in these cases have not been further identified [75, 76, 80].

Zooxanthellae, an integral component of phytoplankton, play a vital role in sustaining the coral reef ecosystem, where dinoflagellates are widely distributed, encompassing both phototrophic algae and heterotrophic predators [81, 82]. Many dinoflagellates are known to harbor various parasites, and photosynthetic dinoflagellates themselves occasionally act as endosymbionts in various marine plankton, including invertebrates, foraminiferans, cnidarian, with a few examples involving ciliates [23, 82, 83].

**Figure 2:**
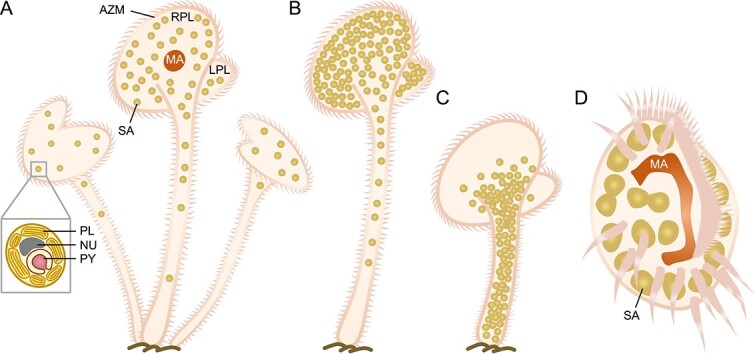
**Ciliates and symbiotic zooxanthellae.** (A) Illustration of a benthic cluster of *Maristentor dinoferus* with endosymbiotic zooxanthellae. (B, C**)** Diurnal changes in the positioning of symbiotic algae in response to the host cell’s shape-shifting. During daylight hours (B), the host cell’s stalk and cap are fully extended, with algae gathered at the cap and stalk peripheries to maximize exposure to light. Conversely, at night (C), the host cell’s stalk contracts noticeably, promoting most intracellular algae to relocate to the stalk. (D) *Euplotes uncinatus* with symbiotic zoochlorellae. The algae exhibit a more elliptical shape compared to those in *M. dinoferus*, and instances of dividing algae cells have been observed. PL, plastid; NU, nucleus; PY, pyrenoid; AZM, adoral zone of membranelles; LPL, left peristomial lobe; RPL, right peristomial lobe; MA, macronucleus; SA, symbiotic algae.

The association of the ciliate *Paraeuplotes tortugensis* with zooxanthellae was first reported in Tortugas, Florida [84]. However, the initial description lacked conclusive evidence based on endosymbionts; instead, it focused on zooxanthellae found in corals [85]. The nature of the relationship, whether the algae are symbionts or food, remains uncertain [86]. *Maristentor dinoferus* is the first unambiguous example of a ciliate with zooxanthellae as symbionts. Ultrastructural and molecular data revealed that the dinoflagellate, as the endosymbiont, belongs to the genus *Symbiodinium* of phylotype clade C [85]. This ciliate was discovered inhabiting coral reefs in Guam, Mariana Islands, with a trumpet shape resembling Stentoridae but phylogenetically related to Folliculindae [85, 87]. Each ciliate cell contains 500–800 dinoflagellates (Fig. 2A) [85]. The dinoflagellates, exhibiting a day-and-night variation rhythm, disperse in the cap of the cell during the day and migrate into the stalk at night, following the host’s shape changes (Fig. 2B, C) [85]. The presence of mycosporine-like amino acids (MAAs), metabolites known to mitigate UV radiation damage, has been observed in *M. dinoferus* with dinoflagellate symbionts [88]. Although the detected MAAs are similar to those found in other dinoflagellate symbionts, there is no conclusive evidence demonstrating that *Symbiodinium* of clade C can synthesize MAAs [88]. *Euplotes uncinatus* harboring zooxanthellae was discovered in the same habitat in Guam. Structural analysis indicates these algae are also dinoflagellates (Fig. 2D) [86]. Despite some dinoflagellates being digested by the host, the intact structure of the algae, observable photosynthetic pigments, evident self-division, and the host’s behavior oriented towards photosynthesis all support the speculation that these dinoflagellates serve as symbionts rather than ingested food [86].

In contrast to the benthic ciliates mentioned earlier, the calcifying ciliate *Tiarina* sp., living in the surface open ocean, was discovered to harbor intact symbiotic *Symbiodinium* endosymbionts [89]. In this scenario, the host *Tiarina* sp. benefits from nutrient sources limited in the open ocean, facilitating its growth and proliferation. Moreover, the symbiotic *Symbiodinium* provides additional advantages such as promoting calcification and protecting against ultraviolet radiation [89].

### *Mesodinium* and cryptophyte: Karyoklepty

Cryptophytes are ubiquitous unicellular **plankton**, predominantly characterized by the presence of permanent chloroplasts generated from secondary symbiosis between a unicellular host and algal symbionts [90, 91]. As primary producers in aquatic food webs, cryptophytes are considered valuable prey for both **heterotrophic** and mixotrophic protists, such as the ciliate genus *Mesodinium*.

The ciliate genus *Mesodinium* exhibits a wide distribution across freshwater, marine, and estuarine environments. Apart from the entirely heterotrophic *M. pupula* and *M. pulex*, other *Mesodinium* species are mixotrophic, ingesting cryptophyte algae and sequestering their plastids, effectively functioning as reduced endosymbionts to achieve phototrophy [92–95]. However, this symbiotic relation is transient and unstable, as ciliate hosts have to regularly obtain fresh cryptophyte prey to replace aging plastids [94, 96–98]. *Mesodinium* species demonstrate the ability to capture cryptophytes from various genera, although not all prey contributes equally to their growth. The observed prey preferences or specificity may reflect varying degrees of evolutionary adaptation to phototrophy within the genus *Mesodinium* [98, 99].

Wild *M. chamaeleon* and *M. coatsi* broadly harbor plastids containing phycocyanin [100, 101], yet they can utilize both red and green cryptophyte species, such as *Chroomonas*, *Hemiselmis*, *Storeatula*, and *Rhodomonas* species [98, 99, 102, 103]. When prey is abundant, *M. coatsi* can replace existing plastids with newly ingested ones within 4 days, regardless of prey type [104]. *M. chamaeleon* demonstrates higher growth rate over longer term when feeding on red cryptophytes, indicating that the origin of plastids may influence the intensity and longevity of the photosynthesis process [102]. Degradation of photosynthetic pigments and plastids necessitates ciliates to replenish their stocks, as there is no evidence of the host’s capacity to synthesize photosynthetic pigments [98, 102]. Replenishing plastid not only restore photosynthetic function but also provides equivalent heterotrophic benefits for growth [98].

**Figure 3:**
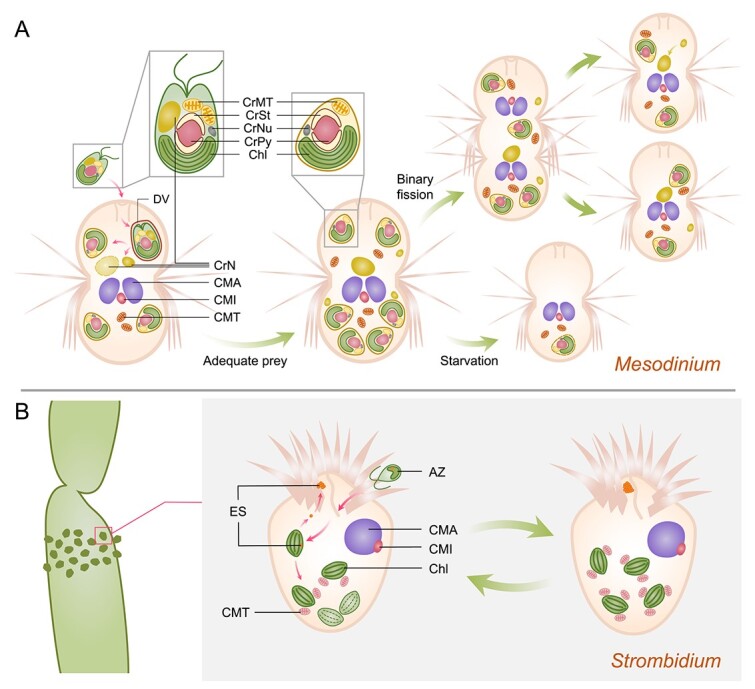
**Karyoklepty and kleptoplasty in *Mesodinium* and *Strombidium*, respectively.** (A) Karyoklepty in the *Mesodinium*-cryptophyte association. Cryptophyte cells, ingested through a cone-shaped oral apparatus, are packaged in digestive vacuoles. Subsequently, the algae lose cytomembranes and nuclei, transforming into organelle complexes containing plastids, cryptophyte mitochondria, pyrenoid, starch granules, nucleomorph, and cytoplasm. These complexes are enveloped by a host vacuole membrane and two ER membranes in the host cytoplasm. Retained cryptophyte nuclei, one of which may relocate to the center of the host cell, undergo significant enlargement and maintain transcriptional activity. After long-term starvation, reserved cryptophyte plastids and nuclei are eventually degraded. During host cell division, cryptophyte plastids are inherited by both daughter host cells, while the central symbiotic nucleus, inherited by only one daughter cell, while another daughter cell may invoke the previously remained prey nucleus. (B) Chloroplasts sequestration in *Strombidium*. *Strombidium* gather around the reproductive part of macroalgae, sequestering chloroplasts and eyespots from zoospores. Stored in host cells for photosynthesis, chloroplasts may have connections with host mitochondria. Eyespots accumulate at the anterior end of the cell, and older chloroplasts are periodically replaced by newly obtained ones. CrMT, cryptophyte mitochondrion; CrSt, cryptophyte starch; CrNu, cryptophyte nucleomorph; CrPy, cryptophyte pyrenoid; Chl, chloroplast; DV, digestive vacuole; CrN, cryptophyte nucleus; CMA, ciliate macronucleus; CMI, ciliate micronucleus; CMT, ciliate mitochondrion; AZ, algae zoospores; ES, eyespots.

The *Mesodinium rubrum/Mesodinium major* complex species have been extensively studied due to their ability to form conspicuous, nontoxic red tides in coastal, fjord, and estuarine ecosystems [105–107]. Despite feeding on various cryptophytes, these ciliates selectively utilize plastids bearing phycoerythrin from *Teleaulax*, *Plagioselmis*, and *Geminigera* cryptophyte species, exhibiting a preference for *Teleaulax amphioxeia* [96, 99, 100, 105, 108]. Recent studies have demonstrated that ciliates can exchange plastids among cryptophyte species, as long as they are available [96]. These ciliates ingest cryptophyte cells, retaining their organelles, including plastids, and thus resemble reduced endosymbionts (Fig. 3A) [94, 96, 109–111]. The photosynthesis carried out by the plastid complexes can fulfill up to 98% of the carbon demand in *M. rubrum*, allowing for a high propagation capacity even at low concentrations of prey organelles [112–114].

To sustain and prolong the biochemical function of plastid complexes, the nuclei of cryptophytes are sequestered and reserved separately in the host cytoplasm (Fig. 3A) [94, 109, 110, 112]. Earlier studies suggested that the enlargement of the central prey nucleus was a result of the fusion of multiple initially sequestered small prey nuclei [109, 115]. However, a later study by Kim et al. [112] revealed that the expansion is not the consequence of nuclei fusion, as the central prey nucleus was observed to enlarge without the availability of other prey nuclei. The other separated prey nuclei are stored at the periphery of the host cell, to be digested later or as possible standbys [112]. Each central prey nucleus has to regulate an average of eight cryptophyte plastids originating from different cryptophyte cells [94]. This symbiotic relationship, exploiting both the plastids and a prey nucleus for photosynthesis, is termed “karyoklepty” [94]. The symbiotic nucleus exhibits sustained transcriptional activity for days before being replaced by other existing prey nuclei [94]. Depletion of cryptophyte nuclei usually results in the incapacity of plastids to divide, leading to a higher frequency of incomplete or abnormal structures, ultimately affecting the number and biosynthetic activity of organelles [94, 109].

In the absence of prey, the central prey nucleus undergoes further enlargement, prompting the starved host *M. rubrum* to initiate cell division, accompanied by the division of sequestered prey plastids [109–112]. The symbiotic consortium maintains its bioactivity for several weeks until the eventual degradation of plastids, posing a potential threat to host survival (Fig. 3A) [94, 108, 111]. After sequestration, cryptophyte genes related to photosynthesis, as well as the biosynthesis of various amino acids and vitamins, are upregulated. This suggests that the sequestration of prey organelle machinery also preserves anabolic potential [108, 115, 116]. The marked up-regulation is deemed necessary for the sole symbiotic nucleus to manage the numerous plastids within the host cell [115, 116]. However, there is an absence of light-dependent transcriptional regulation in free-living *T. amphioxeia* genes [108]. Meanwhile, genes involved in cellular motility are down-regulated, likely attributable to the unnecessary motor activity post-capture [115]. The regulatory pattern of symbiosis expression has been hypothesized to be related to epigenetic control, as multiple genes involved in DNA methylation are highly up-regulated. This suggests potential epigenetic modifications to cryptophyte genes, although further verification is needed [115]. When the host *M. rubrum* is satisfied with prey, genes encoding active transmembrane transporters are upregulated, whereas they are downregulated under starvation conditions [108].

### Intracellular chloroplasts sequestration: kleptoplasty

The phenomenon of impermanent intracellular retention of chloroplasts, known as kleptoplasty, represents another form of mixotrophic behavior observed in ciliates. Ciliates exhibit a diverse range of algae capture, wherein chloroplasts are sequestered, while the remaining parts of algal cells undergo substantial digestion. The chosen chloroplasts remain temporarily sequestered in the ciliate cytoplasm for several days, exhibiting photosynthetic activity and fulfilling their imposed role of supplying carbohydrates and oxygen to the host ciliates [117–119]. In contrast to the phenomenon of karyoklepty observed in *M. rubrum*, there have been no reports of retained algal nuclei, and the sequestered chloroplasts are incapable of division. The acquired phototrophy enhances the growth rate of host ciliates, providing significant competitive advantages over other heterotrophic plankton, particularly in environments with limited oxygen or nutritional resources [120, 121].

**Oligotrich ciliates** that retain plastids play a crucial role in coastal ecosystems during the spring and summer seasons [25, 119, 122–124]. It has been estimated that ~30–40% of oligotrich ciliates in marine environments bear isolated chloroplasts obtained from phytoplankton prey, indicating their mixotrophic nature [120, 123–125]. The overall abundance of oligotrich cells shows a positive association with chlorophyll concentration [120]. Most mixotrophic oligotrich ciliates graze on nanoplankton and selectively acquire chloroplasts, while some marine species exhibit a preference for unicellular **zoospores** of macroalgae [126–128].

The genus *Strombidium*, for example, has been reported to harbor sequestered chloroplasts and **eyespots** from green macroalgae Ulvophyceae in their natural environment (Fig. 3B) [127–130]. These ciliates tend to aggregate around the reproductive tissues of macroalgae, where gametes or zoospores possessing eyespots are abundant (Fig. 3B) [127–130]. *S. oculatum*, for example, exhibits a free-swimming state with positive **phototaxis** during low tide, shifting to negative phototaxis as the tide rises, followed by ciliate **encystment** [127–129]. This phototaxis with tidal adaptability guides the ciliate to suitable microhabitats during tides and is believed to be related to the ciliate’s acquisition of eyespots from algal gamete zoospores, which undergo changes in phototaxis after syngamy [127–129]. Furthermore, *S. stylifer* is speculated to be an obligate mixotroph, as it shows remarkable growth under conditions of adequate illumination with limited food, but deteriorates in darkness [128]. *S. rassoulzadegani*, recommended as an experimental model species for studying chloroplast retention in oligotrichs due to its ease of cultivation and wide distribution, can utilize chloroplasts from various sources, including several species in *Ulvophyceae*, *Dinophyceae*, *Chlorophyceae*, *Haptophyceae*, and *Cryptophyceae* [130–133]. The chloroplasts isolated and retained in the ciliate cytoplasm are observed to be devoid of any vacuolar membrane, presenting in diverse shapes [132]. Although *S. rassoulzadegani* thrives in the light, it can sustain a completely heterotrophic life by feeding on *Tetraselmis chui* in darkness, exhibiting its robust nutritional plasticity and viability [132].

While no instances of algae nuclei retention have been documented within the genus *Strombidium* thus far, a noteworthy discovery has been made regarding *Strombidium basimorphum*. Following the ingestion of the cryptophyte *Teleaulax amphioxeia*, the genetic material of the prey nuclei, nucleomorphs, and ribosomes has been identified in *S. basimorphum* for a period exceeding 5 days [134]. Additionally, transcripts originating from the prey have persisted for 4 days [134–136]. This observation suggests the likelihood of a relatively sustained active transcription of the prey, offering a potential explanation for the continued survival of chloroplasts within the host cell.

Other plastid-retaining marine oligotrichs can be found in genera *Tontonia* and *Laboea*. *L. strobila* is an obligate mixotroph, relying on both plastid photosynthesis within and external food sources for sustenance [118, 137]. Plastids in *L. strobila* were found to possess pigments chlorophyll and phycoerythrin, similar to those in *M. rubrum* [119]. *T. appendiculariformis* harbors brownish plastids believed to originate from specific chromophyte algae species [126]. Each plastid is encapsulated by three membranes, with the outermost membrane presumed to be provided by the host ciliate and possibly linked to the ciliate’s endoplasmic reticulum (ER) [126]. In contrast, in *L. strobila*, the plastids lack surrounding membranes, although they are generally positioned close to the host ER and mitochondria [118].

### Endobacteria of *Euplotes*: Necessary or secondary

**Figure 4:**
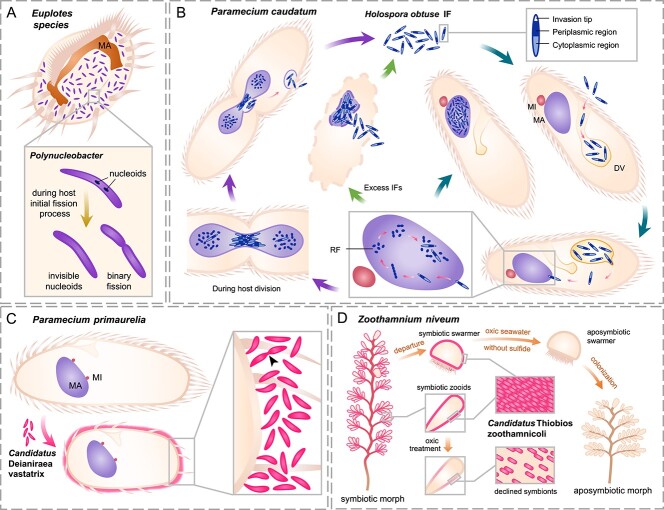
**Illustration of ciliate-bacteria symbiosis.** (A) The obligate symbiotic bacteria *Polynucleobacter* colonize the cytoplasm of *Euplotes* in abundance. During the initial stage of host cell division, the dense nucleoids of symbiotic *Polynucleobacter* turn invisible, and some bacteria may divide at the same time. (B) Infection process of *P. caudatum* by *H. obtuse*. The infectious form (IF) of *H. obtuse*, characterized by straight clavate, sigmoid or arcuate shapes, consists of three parts: the invasion tip, an enlarged periplasmic region, and a condensed cytoplasmic region. Upon encountering aposymbiotic *P. caudatum* cells, IFs enter the host DV. Acidification by the host acidosome activates IFs, allowing them to escape the digestive vacuole with the invasion tip. IFs then move towards the target macronucleus, piercing its membrane via the invasion tip. Inside the macronucleus, IFs differentiate into reproductive forms (RFs), continuously dividing until host cells experience starvation or protein synthesis suppression. Some RFs may revert to IFs. Green arrows indicate that excessive IFs in the macronucleus may inhibit host cell division, potentially leading to host death and freeing the symbiotic bacteria. During host cell binary fission, *H. obtusa* IFs tend to gather at the central connecting site of the dividing macronucleus, and are subsequently released from the host cell. RFs, with a strong affinity to host chromatin, remain in both halves of the macronucleus for the next generation. (C) Ciliate *P. primaurelia* that infected by “*Ca*. Deianiraea vastatrix” will shorten in cell shape and lose its cilia. The ectosymbionts gather and cover host cell surface, forming a dense layer, and can undergo cell division (arrowhead). Sometimes the cell tip of bacteria can directly contact with host membrane. (D) The giant colonial *Z. niveum* has densely distributed thiotrophic ectosymbionts “*Ca*. Thiobios zoothamnicoli”. The thiotrophic bacteria significantly declined under oxic treatment without sulfide. After enough growth, macrozooids leave the colony and develop into dispersible swarmers, which may gradually lose ectosymbionts during long-term sulfide depletion. Finally, the aposymbiotic swarmer settle down and grow into aposymbiotic colony with fewer branches and wider structure than the symbiotic population. MA, macronucleus; MI, micronucleus; DV, digestive vacuole; IF, infectious form; RF, reproductive form.

The **spirotrich ciliate***Euplotes*, prevalent in both freshwater and marine environments, has been a subject of frequent investigation due to its obligate symbiotic association with *Polynucleobacter* spp., primarily *Polynucleobacter necessarius* [138, 139]. Symbiotic *Polynucleobacter* is abundantly present in the cytoplasm of various *Euplotes* species (Fig. 4A), regardless of whether they inhabit freshwater or brackish environments, often in exceptionally high quantities [140, 141]. For example, freshwater *E. aediculatus* has been observed hosting up to 1000 symbiotic *P. necessarius* per cell [138]. This symbiotic relationship is particularly intricate in *Euplotes* species belonging to the clade B group. In this clade, both the host *Euplotes* and the symbiotic *Polynucleobacter* exhibit a tight interdependence, as neither can reproduce or grow independently of the other [41, 138, 142, 143]. A hypothesis suggests that these essential symbiotic bacteria may have assumed functions that were lost in the hosts’ evolutionary history and might even serve as organelles, although specific details remain unclear [142].

Additionally, bacteria from “*Candidatus* Protistobacter” (*Betaproteobacteria*) and “*Candidatus* Devosia” (*Alphaproteobacteria*) can function as essential symbionts in some strains of *Euplotes* clade B, for instance, *Euplotes woodruffi* POH1, *E. eurystomus* EM and *E. octocarinatus* FL(12)-VI [141, 144–146]. However, they are often displaced by *Polynucleobacter*, which appears to be prevalent in the majority of strains in nearly all sequenced *Euplotes* species of clade B, including freshwater species like *E. octocarinatus*, *E. daidaleos*, *E. eurystomus*, *E. aediculatus*, and brackish-water species like *E. woodruffi*, *E. harpa* (= *E. platystoma*) [41, 138, 140, 141, 144, 146]. Furthermore, numerous bacteria can lead a symbiotic or parasitic existence, predominately as secondary/tertiary symbionts, in *Euplotes* of clade B, particularly belonging to *Alphaproteobacteria*, *Betaproteobacteria* and *Gammaproteobacteria* [41, 147, 148]. Some bacteria from the family *Candidatus* Midichloriaceae have been found in *E. harpa* and *E. woodruffi*, coexisting with *Polynucleobacter* [147, 148]. *E. aediculatus* and *E. octocarinatus* may experience additional infections by bacteria in Alphaproteobacteria and Gammaproteobacteria [149–151]. While it is assumed that the essential symbiont *Polynucleobacter* may play a mysterious role during the colonization process of secondary symbionts, no confirmed information regarding related mutual interactions has been established to date.

While rare, species out of clade B have also been observed to host bacterial endosymbionts. For instance, the marine species *E. magnicirratus* in clade A harbors “*Candidatus* Devosia euplotis” [152, 153]. Notably, a strain of bacteria belong to the genus *Francisella*, renowned for its pathogenic properties, was initially isolated from the marine species *E. raikovi* (clade C) and later classified as *Francisella endociliophora* [154, 155].

The original endosymbiotic *Polynucleobacter* spp. can be removed from host cells through antibiotic treatment [143, 156, 157]. Subsequent re-infection experiments are typically conducted through microinjection rather than the host’s **phagocytosis** [143, 156]. Successful re-infection occurs only when symbiotic bacteria are injected into their original host species, indicating a high specificity of colonization or mutual dependence [143]. In addition, exposing aposymbiotic *Euplotes* cells to homogenates of ciliate cells containing endosymbionts can result in a limited quantity of cell re-infection, although the outcomes are inconsistent [157]. As the natural infection process remains unspecified, it is speculated that the original *Polynucleobacter* bacteria may have entered the *Euplotes* cell cytoplasm and fortuitously established themselves as endosymbionts. The host-symbiont group may have undergone co-evolution, leading to various stages that manifest as highly complex and infrequent cross-infection between non-original partners [143, 156]. Moreover, **phylogenetic analysis** indicates that the sequenced *Polynucleobacter* species have independently evolved from distinct origins [158].

### Intranuclear bacteria: *Holospora* in *paramecium*

While most symbionts prefer colonizing in the host cytoplasm, certain species exhibit a particular affinity for the host nuclei, a strategic compartment to avoid lysosome threats [19, 159]. Intranuclear bacteria of the genus *Holospora* (*Alphaproteobacteria*, *Holosporales*) and some *Holospora*-like bacteria (HLB) are extensively studied, primarily within the ciliate *Paramecium*, residing in both types of nuclei, **macronucleus** and **micronucleus**.

*Holospora* and HLB symbionts demonstrate species and nuclear specificity, with each species of *Holospora* spp. typically infecting a specific type of nucleus in a limited number of host species. For example, *Holospora obtusa*, a well-studied intranuclear bacterium, exclusively invades the macronucleus of *Paramecium caudatum*, while the host micronucleus typically harbors *Holospora elegans*, *H. undulata*, or *H. recta* [160, 161]. Similarly, *Holospora curviuscula* and *H. acuminata* infect the macronucleus and micronucleus of *P. bursaria*, respectively [162]. In contrast to those with strict host species specificity, *H. caryophila* has been found within the macronucleus of various hosts such as *P. triaurelia*, *P. tetraurelia*, *P. biaurelia*, *P. jenningsi*. [163]. Interestingly, phylogenetic analysis reveals that symbiotic *Holospora* species clusters are related to the species of hosts rather than the types of nuclei they inhabit [162, 164].

Throughout the intricate infection life cycle (Fig. 4B), two distinct forms of *Holospora* spp. alternately emerge: the infectious form (IF) and the reproductive form (RF) [19, 165, 166]. A crucial step in the invasion process is the escape of bacteria from the host’s digestive vacuole [167]. Taking the *P. caudatum*–*H. obtusa* interaction as an example, IFs undergo acidification and activation facilitated by the fusion of the host acidosome and digestive vacuole. Subsequently, they exit the vacuole using an invasion tip structure and secrete tip-specific 89-kDa proteins [167–170]. Once in the host cytoplasm, IFs are assisted and supported by actin aggregation, facilitating movement towards the target nucleus [171]. Subsequently, IFs penetrate the nucleus membranes through the invasion tip, leaving behind the 89-kDa proteins and giving rise to the 63-kDa proteins [168, 172]. The IF-specific 63-kDa proteins, subsequently renamed periplasmic region protein 1, are secreted into the host macronucleus, where they can bind to host macronuclear DNA [172, 173]. Once inside the nucleus, IFs differentiate into RFs, primarily dedicated to propagation [174]. As *P. caudatum* cells undergo binary fission, *H. obtusa* IFs can be expelled from the host cell, retaining viability in the external environment for a limited period, during which they attempt to infect other potential hosts [175–177].

*Holospora* spp. were initially considered as parasitic bacteria, as an excess of IFs in the macronucleus could hinder host cell division, potentially leading to the host’s death, and subsequently freeing the symbiotic bacteria (Fig. 4B). Furthermore, *H. undulata* has been observed to increase the mortality of host *P. caudatum* during food deficiency [177]. Despite the potential harm caused by an overabundance of bacteria, *Paramecium* hosting appropriate symbiotic *Holospora* can gain enhanced resistance to environmental stress. *P. caudatum* with macronucleus-specific *H. obtusa* or micronucleus-specific *H. elegans* exhibits a stronger heat-shock resistance and higher expression of the heat-shock protein gene hsp70 when the intranuclear bacteria are in the RFs state [178, 179]. Additionally, symbiotic *Holospora* may contribute to the osmotic-shock resistance of the host [19, 167].

### Ciliates and endobacteria: Reduced genome and metabolic interaction

The confined living spaces, limited population sizes, and unique habitats have subjected endosymbiotic bacteria to specific selective pressures, resulting in streamlined genome evolution characterized by varying degrees of metabolic gene reduction and genome size shrinkage [39, 158, 164, 180]. It is asserted that free-living bacteria typically have genome sizes ranging from 2 to 10 Mb, a range notably diminished to less than 1.5 Mb in the majority of bacterial obligate endosymbionts [181]. The obligate endosymbiotic *Polynucleobacter* observed in *Euplotes* belongs to subcluster C (PnecC) of the *Polynucleobacter* cluster, which also includes some obligate free-living strains [181]. Nevertheless, the genome sizes of endosymbiotic strains (1.7 ~ 1.8 Mb) are significantly reduced compared to their free-living relatives (2.1 ~ 2.5 Mb), accompanied by a notable decline in coding DNA and an increase in **pseudogenes** [181, 182].

Many members of *Alphaproteobacteria* within the orders *Holosporales* and *Rickettsiales* are recognized as symbionts of protists. Initially identified as part of *Rickettsiales sensu lato* and considered sister to *Rickettsiales* in early phylogenetic studies, subsequent analyses employing diverse methodologies have placed *Holosporales* within the order *Rhodospirillales* [183]. The sequenced genomes of *Holospora* range from 1.27 to 1.72 Mb, with a GC content ranging from 35.2% to 37.6% [47, 160, 164]. Despite *Rickettsiales* already known for possessing gene-poor genomes, *Holosporales* exhibit further reduction in metabolic genes, rendering them incapable of normal growth or reproduction outside their hosts [164].

**Fig. 5:**
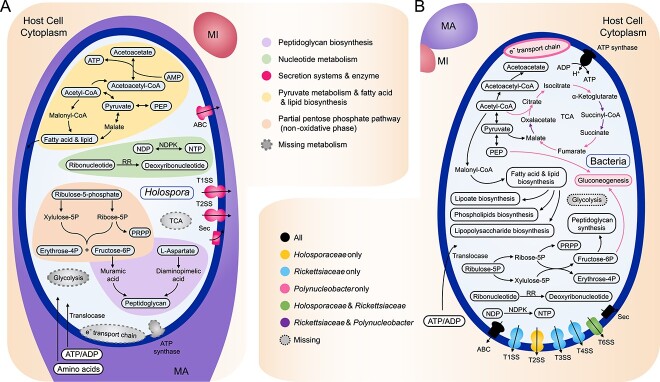
**Predicted metabolic pathways in the symbiotic bacteria.** (A) Schematic representation of the main predicted metabolic features of *Holospora*. The color signs on the right refer to different metabolic pathways. (B) Comparison of the existence of main metabolic pathways among *Holosporaceae*, *Rickettsiaceae*, and *Polynucleobacter*. The color signs on the left refer to the existence condition of different bacteria groups. MA, macronucleus; MI, micronucleus; PEP, phosphoenolpyruvate; NDPK, nucleoside-diphosphate kinase; RR, ribonucleotide reductase; TCA, tricarboxylic acid cycle; PRPP, phosphoribosyl pyrophosphate; T1SS, type I secretion system; T2SS, type II secretion system; T3SS, type III secretion system; T4SS, type IV secretion system; T6SS, type VI secretion system.

The genomes and metabolic pathways of symbiotic bacteria exhibit lineage-specific plasticity, influenced by varying degrees of host dependence and host-associated lifestyles [184]. This reduction in metabolic pathways is particularly evident in obligate endosymbiotic bacteria such as *Polynucleobacter*, *Holosporaceae*, and the well-studied symbiont *Rickettsiaceae*, affecting carbohydrate, lipid, amino acid, nucleotide, and energy metabolism, as well as secretory systems (Fig. 5) [38, 164, 182]. The limited biosynthesis necessitates endobacteria’s reliance on host-imported sources of carbon, nitrogen, and other metabolic products [182]. Moreover, symbiotic *P. necessarius* has lost the assimilatory ability for absorbed nitrate, sulfur, or sulfate but retained most of the amino acid biosynthesis [182]. Intriguingly, all *Holospora* species can synthesize ribonucleotide reductases, converting ribonucleotides into deoxyribonucleotides, making nucleotides or ribonucleotides the main energy source observed thus far [164]. This aligns with the nuclear parasitic characteristic of *Holospora* spp. Nevertheless, *Holospora* spp. have completely lost the type VI secretion system, a feature prevalent among other *Holosporales*, suggesting a correlation between the type VI system and intracytoplasmic lifestyle [19]. The endosymbiotic “*Candidatus* Fokinia solitaria” (*Rickettsiales*, “*Candidatus* Midichloriaceae”), living in *Paramecium* sp., possesses an extremely reduced genome (~837 kb) compared to its sister species, having lost genes encoding flagellar proteins, lipopolysaccharide biosynthesis enzymes, and components of the Krebs cycle [185]. Similarly, the symbiont of *P. polycaryum*, “*Candidatus* Gromoviella agglomerans” (*Holosporales*, *Holosporaceae*), exhibits an even smaller genome (<600 kb) with severely limited biosynthetic and energy metabolism capabilities, albeit retaining functional membrane transporters and secretion systems [186]. These bacteria heavily rely on the host for survival, and their aggregation likely contributes to incomplete host cell division [186]. Moreover, prolonged residence within the host cytoplasm leads to a gradual decline in symbiotic reactions and resistance to unfavorable environmental factors [182].

Some symbionts have obtained specific functions via horizontal gene transfer or retained valuable biosynthetic pathways in early evolutionary lineages to enhance their survival. For instance, the endobacteria of *P. tredecaurelia*, “*Candidatus* Sarmatiella mevalonica” (*Rickettsiales*, *Rickettsiaceae*), possesses the complete gene repertoire for the mevalonate pathway, enabling the utilization of host-produced metabolites [187]. Given that the mevalonate pathway is common in eukaryotes but rare among Proteobacteria, and these genes are also found in other bacteria derived from metagenomes, it is considered an example of convergent evolution driven by horizontal gene transfer [187]. Episymbiotic bacteria densely covering the surface of the ciliate *P. primaurelia* have been identified as *Deianiraea*, belonging to *Deianiraeaceae* within *Rickettsiales*. Severe infection by *Deianiraea* can lead to host cell shortening and significant reduction in ciliature (Fig. 4C) [188]. This discovery confirms that episymbionts within *Rickettsiales* possess the richest amino acid biosynthetic capabilities, surpassing other members within *Rickettsiales* [188]. Moreover, *Deianiraea* exhibits a notable two-partner type V secretion system and type II secretion system, both contributing to adhesive effects [188]. The unique extracellular lifestyle and distinctive biosynthetic traits suggest the possibility of an extracellular ancestor within *Rickettsiales* with a complex and diverse metabolism, which subsequently gave rise to other obligately endosymbiotic descendants with reduced biosynthetic complexity in independent sublineages [188].

Unlike the complexity of the external environment, the habitats of endosymbionts inside host cells are notably stable and secure, alleviating the selection pressure on biological functions. Each colony of endosymbiotic bacteria typically originates from a small number of ancestral individuals, engaging in subsequent asexual reproduction within a limited and small population size. The opportunity for recombination between strains of external bacteria is restricted, resulting in reduced genetic diversity and an evolutionary bottleneck driven by **genetic drift** [39, 158, 189]. Unlike most other symbionts, which face challenges due to significantly distant phylogenetic relationships with free-living strains, leading to saturation of synonymous substitutions, *P. necessarius* provides an excellent opportunity for studying genetic drift [158]. Comparisons of non-synonymous and synonymous substitution rates between symbiotic and free-living strains confirm that the genomic reduction observed in symbiotic *Polynucleobacter* is mainly propelled by genetic drift. In this context, mutation rates are more reflective of lineage differences rather than symbiosis-related factors [158]. Complex factors contribute to gene deletion and pseudogene generation [39, 182]. Additionally, the degeneration of DNA repair mechanisms is considered to contribute to the bias of A + T bases in genome composition, resulting in lower energy consumption [39, 189].

### Ciliate-dominated symbiotic relationships

Originally heterotrophic organisms, ciliates primarily acquire nutrition through the direct digestion of prey, including bacteria, fungi, and algae. Their survival is inevitably influenced by factors such as prey availability, competitions from other heterotrophic organisms, and environmental conditions. Seeking any advantageous opportunities for survival, including assistance from other symbiotic organisms, has been an evolutionary strategy retained by ciliates. The establishment of host-dominated symbiotic relationships is typically driven by the ciliate’s desire to expand ecological niches, gain a competitive advantage over rivals, or escaping from predators.

Microorganisms with unique energy metabolism patterns have proven to be attractive symbionts. The associations between ciliates and photosynthetic microbes are influenced by environmental factors such as light and oxygen availability. Aerobic algae with oxygenic photosynthesis offer ciliates additional oxygen and organic matter, sometimes fulfilling the host’s requirement for vegetative growth [12, 23]. In oxygen-deficient environments, ciliates acquire phototrophy from anaerobic primary producers like purple bacteria, which fix inorganic carbon without producing oxygen [190, 191]. Examples include the facultatively anaerobic *Strombidium purpureum*, which harbors purple non-sulfur bacteria for anoxygenic photosynthesis [191, 192]. Another instance is the endosymbiotic purple sulfur bacteria “*Candidatus* Thiodictyon intracellulare”, coexisting with small amounts of green algae *Chlorella* sp., forming a physiologically flexible consortium adaptable to varying light and oxygen conditions [191, 193]. These associations are mainly found in hypoxic but organic matter-rich sediments, where ciliates rely mainly on anoxygenic photosynthesis of purple symbionts, phagocytosis, and fermentation of symbiotic partners [193].

In deep waters where light and oxygen penetration is limited, obligately anaerobic ciliate species harbor endosymbiotic denitrifying bacteria like “*Candidatus* Azoamicus ciliaticola” [194], which possess reduced genomes but retain genes for denitrification. These symbionts efficiently produce and convert energy for the host [194], potentially supplementing or substituting host mitochondria [194]. Additionally, sessile ciliates like *Zoothamnium niveum* inhabit sulfur-rich environments with thiotrophic ectosymbionts “*Candidatus* Thiobios zoothamnicoli” (Fig. 4D) [42, 195, 196]. These symbionts fix carbon and transfer it to the host cell under oxic and sulfidic conditions, but decline in the absence of sulfide, leading to limited host growth and lifespan [197].

The acquisition of unique energy physiological patterns by symbionts provide ciliates with additional nutrient access, reducing dependence on limited food sources and competition with other protists. Symbionts can also serve as occasional food sources during prey scarcity, prolonging host survival [66]. Ciliates hosting symbionts exhibit particular advantages in oligotrophic environments due to lower survival costs. For example, *Limnostrombidium viride* benefits from sequestered chloroplasts and thrives in freshwater ecosystems with limited nutrients but sufficient light [124, 198–200]. The symbiont-bearing ciliates’ increased metabolic activities enable them to exploit and occupy specific ecological niches. For instance, *Perispira ovum* and *Histobalantium natans* preferentially prey on euglenoid flagellates, sequestering their chloroplasts and mitochondria and thriving in low-light anoxic environments [201, 202].

Symbiotic relationships provide stable shelters against predators, variable environments, or potential pathogens [63, 67], with symbionts benefiting from continuous supply of metabolic substrates. However, these relationships are mainly controlled by hosts, which have developed various mechanisms to manage symbiont conditions [203]. Host ciliates optimize the utilization of symbionts through domestication or modification, retaining only useful organelles [12]. Symbiont populations are regulated by host control over intake, consumption, and proliferation. For example, *P. bursaria* can adjust the quantity of endosymbiotic *Chlorella* in response to varying light intensities, ensuring low-cost, high-photosynthate acquisition [203]. Moreover, investigations of acquired phototrophy in *Mesodinium*, *Strombidium*, and *Euplotes* have shown that symbionts can be replaced with external candidates if available [96]. Therefore, in host-dominated symbiotic relationships, the captive symbionts represent an alternative option for hosts, and the development of these survival alliances are primarily driven by the host, ensuring the perpetuation of traits that confer benefits to the host.

## Concluding remarks

The symbiotic events in ciliates offer high-quality model systems for investigating host-symbiont interactions, as ciliates engage their entire bodies in symbiosis. The establishment of phototrophic symbiosis primarily hinges on the host’s need for photosynthate, achieved through various degrees of preservation of algal cellular structures and genetic materials. The photosynthetic products obtained through acquired phototrophy may supplement nutrients acquired from intrinsic phagotrophy, potentially resulting in an elevated growth rate of host cells or survival advantages over other species in challenging environmental conditions. Bacterial endosymbionts exhibit a spectrum of diversity, ranging from mutualistic cooperation to temporary infectious exploitation. The transition from original free-living strains to obligate bacterial endosymbionts occurs due to rapid proliferation rates and specific selective pressures. This evolutionary shift is accompanied by the decline of numerous functional genes to varying extents, with the detailed molecular mechanisms being preliminarily elucidated. These investigations hold promise for studying the ecological functions of symbiosis, exploring physiological cellular interactions, and understanding the evolutionary strategies of eukaryotes.

### Glossary

**Acquired Phototrophy:** The phenomena of organisms gaining photosynthetic ability by utilizing their endosymbiotic phototrophs or isolated photosynthetic organelles.

**Ciliate:** A single-celled organism belonging to the protozoan phylum Ciliophora and possessing cilia on the cell surface.

**Digestive Vacuole (DV):** A membrane sac in the cell that envelops and digests food using lysosomal enzymes.

**Encystment:** The process by which some organisms form a protective dormant cyst to cope with adverse environmental conditions.

**Eyespot:** A pigmented organelle in some single-celled phototrophic organisms that play a role as a light receptor.

**Genetic Drift:** The random change in the frequency of a particular allele in a small and isolated population, which may result in the flourishing or disappearance of related genetic traits.

**Heterotrophy:** An organism (termed heterotroph) survives on nutrition originating from other organisms.

**Macronucleus & Micronucleus:** Nuclear dimorphism is one of the essential features of ciliates, where the somatic and germline genomes are separated into macronucleus and micronucleus, respectively. The macronucleus is relatively large and is responsible for vegetative growth, while the micronucleus controls the reproductive processes.

**Mixotrophy:** Free-living protozoans engage in both heterotrophy and autotrophy.

**Oligotrich Ciliates:** A group of ciliates belonging to the order Oligotrichida, characterized by conspicuous adoral ciliature and bristle-shaped or cirri-shaped cilia.

**Perialgal Vacuole (PV):** A membrane sac housing alga in the host cell that differentiates from the host digestive vacuole.

**Phagocytosis:** The process by which living cells ingest whole particulate substances into the cell. In some single-celled protists, phagocytosis is used to acquire food.

**Phototaxis:** The organism’s behavior of moving toward or away from the direction of light, divided into positive and negative phototaxis.

**Phylogenetic Analysis:** An analysis that attempts to investigate the evolutionary relationships among biological entities.

**Plankton:** Small organisms that are weak in swimming, drifting about in marine or freshwater, including phytoplankton (plants) and zooplankton (animals).

**Pseudogene:** A nonfunctional copy of genomic DNA that closely resembles gene but does not encode a protein.

**Spirotrich Ciliates:** A group of ciliates belonging to the class Spirotrichea.

**Symbiosis:** The association of two or more different species living together, and the members involved are called symbionts. Endosymbiosis is the relationship of an organism living within another organism of a different species.

**Zoospore:** The reproductive cell with one or more flagella, produced by some algae.

## Data Availability

Data sharing not applicable to this article as no datasets were generated or analyzed during the current study.
